# Gene Editing and Alzheimer's Disease: Is There Light at the End of the Tunnel?

**DOI:** 10.3389/fgeed.2020.00004

**Published:** 2020-06-03

**Authors:** Mikhail Stepanichev

**Affiliations:** Laboratory of Functional Biochemistry of the Nervous System, Institute of Higher Nervous Activity and Neurophysiology, Russian Academy of Sciences, Moscow, Russia

**Keywords:** Alzheimer's disease, CRISPR/Cas9, genome editing, amyloid-β, brain

## Abstract

Alzheimer's disease continues to be a fatal, incurable neurodegenerative disease, despite many years of efforts to find approaches to its treatment. Here we review recent studies on Alzheimer's disease as a target for gene therapy and specifically, gene editing technology. We also review the opportunities and limitations of modern methods of gene therapy based on the CRISPR editing system. The opportunities of using this approach for modeling, including cellular and animal models, studying on pathogenesis and disease correction mechanisms, as well as limitations for its therapeutic use are discussed.

Alzheimer's disease (AD) is the most common age-related form of dementia. Despite extensive efforts, the understanding of AD pathogenesis continues to be elusive. Furthermore, the search for possible therapeutic targets is mostly unsuccessful and the products of several pharmaceutical giants developed for AD treatment failed in clinical trials. The only treatment available for AD for many years has been the use of drugs, mainly inhibitors of acetylcholine esterase (AChE), directed to boosting cholinergic transmission in the brain of patients. It is known that early symptoms of AD are associated with cognitive impairments probably because of cholinergic dysfunction. The cholinergic hypothesis of age-related memory impairments was suggested in the 1980-s (Bartus et al., 1982) and now it is fully recognized by most researchers in the field. Degenerative alterations in basal forebrain cholinergic neurons (BFCN) serve as a basis of memory disturbances, specifically, in the early stages of AD development. However, the factors which cause cholinergic deficit in AD are not known. There are not any specific mutations in the genes encoding proteins directly involved in cholinergic transmission. Genome-wide association studies (GWAS) have revealed very complex genetic modifications related to AD etiology, but none of observed associations was related to the early cholinergic insufficiency. On the contrary, mining GWAS data for biologically meaningful information to a late-onset AD GWAS dataset has demonstrated a significant overrepresentation of association signals in pathways related to cholesterol metabolism and the immune response (Jones et al., 2010). The only non-immune and non-lipid related process detected in the study was cholinergic synaptic transmission.

We have to mention that not only BFCNs, primarily in the nucleus basalis of Meynert (NbM), degenerate at the early stages of AD. Neurodegeneration is also observed in the entorhinal cortex (EC), hippocampus and locus coeruleus (LC). In all three areas, neuronal loss becomes detectable already at preclinical stages and clearly manifests at prodromal AD/mild cognitive impairments (MCI). At more advanced AD, cell loss is most pronounced in the NbM > LC > layer-II EC. However, during early AD, the extent of cell loss is fairly balanced between all three areas without clear indications for a preference of one area (Arendt et al., 2015). Furthermore, a glutamatergic imbalance in the neocortex correlates with AD severity (Francis et al., 1993). In sporadic AD, glutamate concentration was shown to fall since it may serve as a substitute for lacking glucose in the beginning of the disease. In contrast, the glutamate receptor density was found to be much less involved indicating excessive activation of the glutamatergic system particularly via the NMDA receptor, mediating endogenous excitotoxicity (Riederer and Hoyer, 2006). This may explain the symptomatic effectiveness of the NMDA antagonist memantine, the second drug actively used for the treatment of moderate-to-severe AD (Howard et al., 2012).

The pathogenesis of several familial forms of AD is relatively well-studied. Mutations in the genes encoding presenilins *PSEN1* and *PSEN2* and the amyloid-β precursor protein (*APP*) are associated with early-onset AD whereas mutation in the gene encoding apolipoprotein E ε4 (*APOE* ε*4*) is associated with late-onset AD. However, genetic factors are responsible for no more than 10% of all early-onset AD cases, and only up to 25% of all late-onset AD cases are associated with the presence of the *APOE* ε*4* mutant allele (György et al., 2016; Verheijen and Sleegers, 2018). In the case of late-onset AD, the presence of a known allele of AD vulnerability does not provide much help in clarifying our understanding of the pathogenesis of the disease. To date, AD pathogenesis seems to be related to more than 50 mutations (Sims et al., 2020) and whole genome associated studies demonstrate that many gene loci are responsible for the onset of the disease.

Several years ago, it seemed that gene therapy was the most promising approach to the treatment of neurodegenerative pathologies, as it allows curing of inherited or non-inherited diseases via gene transduction into patients' cells in order to directly modify genetic defects or add new functions to them. In the field of AD therapy, the development of the gene therapy strategy was again based on the cholinergic hypothesis. It is well-known that an insufficiency of nerve growth factor (NGF) may promote the development of a cholinergic deficit (Schaub et al., 2002). Uncontrollable secretion and distribution of NGF in the brain is associated with adverse side effects; therefore, autologous fibroblasts preliminarily transduced with vectors based on recombinant lentivirus or adeno-associated virus (rLV and rAAV, respectively), which carry the *NGF* gene, were used in clinical trials. In 2005, the results of phase I of clinical trials using the *ex vivo*-gene therapy approach in a group of eight patients with mild AD (stage V/VI according to the Braak scale) were reported (Tuszynski et al., 2005). This group of patients demonstrated some improvement in cognitive functions, brain metabolism, and the morphological state of cholinergic neurons. The data from a pathomorphological study of the delayed consequences of the therapy were published later (Tuszynski et al., 2015). In this study, two more patients with direct vector injection into the caudal part of the NbM were added to the previous group. In all patients, the response of degenerating neurons to trophic treatment was observed in the form of sprouting, hypertrophy, or the activation of intracellular signaling. However, no pathological alterations were found. NGF-induced sprouting might be maintained for more than 10 years. The authors concluded that additional testing of this gene-therapy approach for its introduction into clinical practice would be promising.

Functional genomics allowed substantial improvement in specific tools for experiments and gene therapy. In the present review we shall discuss modern studies directed to the application of these new approaches to create new models which allow us to understand the pathogenesis of AD or to correct the known mutated genes associated with AD.

## Designer Nucleases for Directed Genome Editing

Classical gene transfer using viral vectors results in random integration of specific genes into the genome as well as within cell episomes. In the therapeutic field, the replacement of random integration with targeted gene insertion, or targeted correction, has become a commonly accepted potential solution. In principle, genome editing consists of making a break in double-stranded DNA in a locus where the target gene is located, followed by reparation of this break. Endonucleases are enzymes that cleave DNA via catalysis of the disintegration of phosphodiester bonds within polynucleotide chains. At present, three types of nucleases are the main tools used in experimental studies: zinc finger nucleases (ZFN) (Kim et al., 1996), transcription activator-like effector nucleases (TALEN) (Miller et al., 2011), and clustered, regularly interspaced, short palindromic repeats (CRISPR)/CRISPR-associated system (Cas), i.e., CRISPR/Cas, system (Doudna and Charpentier, 2014).

Each of these genome editing tools consists of endonuclease, which cleaves double-stranded DNA, and a DNA binding domain, which allows endonuclease binding with DNA at a specific site (Chugunova et al., 2016). For ZFN, the precision of binding to a DNA site is determined by the presence of at least three zinc finger proteins and the affinity of the binding increases with the number of DNA-binding zinc fingers, although this, in turn, reduces the activity of the construction as a whole (Shimizu et al., 2011). In order to cut two strands of DNA precisely, the nuclease has to form a dimer. Therefore, to edit a specific site, it is important to use a pair of proteins differing in the composition of zinc fingers.

The other construction type used for editing is based on the application of TALE proteins for DNA binding. In contrast to ZFN, in which each domain of a zinc finger interacts with three nucleotides, one domain of a TALE protein binds one nucleotide. Thus, it is necessary to synthesize a relatively large protein with many domains to provide specific binding of TALEN with DNA target sites (Cermak et al., 2011; Lee et al., 2016). Two DNA chains are cut after the formation of a dimer, which, as in the case of ZFN, necessitates the use of two proteins simultaneously to edit one gene. Both ZFN and TALEN have a similar, relatively low efficacy, but the cytotoxicity and difficulty of construction preparation is lower when TALEN is used (Chugunova et al., 2016).

CRISPR/Cas9 is an element of adaptive immunity of prokaryotes. This system consists of a CRISPR cassette and a Cas9 nuclease. CRISPR cassettes and Cas together provide prokaryotic cell resistance to bacteriophages and plasmids containing protospacers, which are complementary to those in a CRISPR cassette (Zhang et al., 2014; Savitskaya et al., 2016). In this system the Cas9 nuclease forms a double-strand DNA break, and the site of this break is determined by the sequence of a single-chain guide RNA (sgRNA). Of the large spectrum of Cas nucleases, the Cas9 enzyme is the most studied and most widely used. Generation of a site-specific double-strand DNA break is followed by one of two main types of repair: Non-homologous end joining (NHEJ) or Homologous dependent repair (HDR). NHEJ binds free DNA ends at the site of double-strand damage to each other forming small insertions or deletions (indels). The HDR mechanism repairs the DNA break using an additional template to make a copy of the damaged fragment. A sequence of nucleotides of an intact paired chromosome or a sequence of nucleotides introduced from the outside with a plasmid or oligonucleotide may be used as a template. In practice, the first approach is used when it is necessary to turn off the gene, and the second when it is necessary to make a correction of the mutant gene.

In addition, a base-editing approach has been developed that makes it possible to precisely convert one nucleotide to another in DNA or RNA without inducing a double-strand DNA break (reviewed in Molla and Yang, 2019). A combination of catalytically impaired Cas9 variants with different deaminases has yielded diverse base-editing platforms that aim to address the key limitations such as specificity, protospacer adjacent motif (PAM) compatibility, editing window length, bystander editing, and sequence context preference. Because new base editors significantly reduce unintended editing in the genome, they hold great promise for treating genetic diseases, including AD (Park et al., 2017).

The use of sgRNAs is more technologically feasible and cost-efficient as compared to ZFN and TALEN, which require time-consuming customization of DNA binding proteins. CRISPR/Cas9 may be relatively easily packed into one rLV-based vector, and the high efficiency of editing and lower cytotoxicity, at least compared to ZFN (Chugunova et al., 2016), has provided explosive progress in research using genome editing.

## Looking Into AD Pathogenesis With Genome Editing Tools

As mentioned above, AD is a disease probably associated with multiple gene modifications. Only a small portion of patients has familial AD caused by specific mutations in one of three genes, such as *APP, PSEN1*, or *PSEN2*. However, AD pathogenesis is associated with over 50 gene loci that are responsible for AD onset, indicating that AD, specifically late-onset AD, is a disease of multiple components, as supported by pathway analysis (Sims et al., 2020). However, it is important not only to find the genes associated with the disease but also to identify the functions of these genes. The CRISPR system was recently adapted for functional genome-wide screening. Using CRISPR/Cas9 based inhibition or activation of a wide spectrum of genes it is possible to identify relevant determinants of pathological processes, such as for example selective vulnerability of neurons that may lead to possible therapeutic candidates (Kampmann, 2017). This area of research is rapidly developing. Tomita et al. (2017) transduced Cas9-expressing murine neuroblastoma N2a cells with a whole genome targeting gRNA lentiviral library and used the transduced cells for studies on phagocytosis of aggregation-prone amyloid-β (Aβ) protein at a molecular level. They found that genes involved in endocytic machinery and the immune response pathway affected the Aβ uptake. In addition, interactome analysis revealed that the *INPP5D* gene encoding Src homology 2 (SH2) domain, containing inositol polyphosphate 5-phosphatase 1 and *CD2AP* gene encoding CD2-associated protein, which are known as genetic AD risk factors, were mapped in the same protein-protein interaction network with genes that were identified by the screening.

Another study uncovered the cellular pathways controlling prion-like propagation of τ aggregation. CRISPR interference (CRISPRi) screens were performed in a human cell-based model of propagation of τ aggregation (Chen et al., 2019). This revealed that knockdown of several components of the endosomal sorting complexes required for transport (ESCRT) machinery, including charged multivesicular body protein 6 (CHMP6), or CHMP2A in combination with CHMP2B (whose gene is linked to familial fronto-temporal dementia), promote propagation of τ aggregation. These findings suggest that endolysosomal escape is a critical step in τ propagation in neurodegenerative diseases.

Further support to the idea of impaired endosomal functioning in AD was observed in the study by Kwart et al. (2019) who created a panel of isogenic knockin human iPSC lines carrying APP and/or PSEN1 mutations. Global transcriptomic and translatomic profiling revealed that familial AD mutations had overlapping effects on the expression of AD-related and endocytosis-associated genes, including increased Rab5+ early endosome size. These authors found that APP and PSEN1 mutations had discordant effects on Aβ production but similar effects on APP β C-terminal fragments, which accumulated in all mutant neurons. The additional advantage of the genome editing system in this case was the use of a non-overexpression human-based system.

Skin fibroblasts were used for the development of an *in vitro* model system based on CRISPR transcriptional activation analysis of APP and/or BACE1 (Inoue et al., 2017). The increased Aβ level in skin fibroblasts as well as γ-secretase processing defects were revealed in fibroblasts derived from patients with familial AD. This activated CRISPR skin fibroblast model will prove beneficial to probe the role of various genetic modifiers of sporadic AD.

Cholinergic neurotransmission is very important for understanding the mechanism of cognitive decline in AD as well as the most real target for known anti-AD therapy. In order to study in more detail the fate and the mechanism of functioning of cholinergic neurons in the absence of ChAT expression, a model of ChAT knockdown using CRISPR/Cas9 is developing (Stepanichev et al., 2019). Two types of receptors mediate the effects of ACh in the brain, specifically muscarinic (mAChR) and nicotininc (nAChR). It is known that G-protein coupled M1 mAChR is impaired in the neocortex of AD patients and severity of cognitive symptoms in AD is greatly related to the degree of M1/G-protein uncoupling. Activation of M1 mAChR shifts APP metabolism to the non-amyloidogenic pathway and reduces τ hyperphosphorylation via glycogen synthase kinase-3β inhibition, increased ERK activity and potentiation of NMDA receptor (see for review Verma et al., 2018). It has been reported that the interaction between α7 nAChR and Aβ fragments exerts neurotoxic effects through blocking of α7 nAChR that follows the internalization of the fragments. Furthermore, Aβ probably impairs cognitive functions through the nAChR-dependent mechanism (see for review Verma et al., 2018). ACh can also interact with α7 nAChR located on microglia cells and thus, modulate cognitive functions and neuroinflammation (Maurer and Williams, 2017). Despite the importance of ACh receptors, it is very difficult to dissect the function of individual receptor subunits in the brain. The CRISPR/Cas9-based methods were recently applied to overcome this technical gap. Thus, Niwa et al. (2018) have reported that CRISPR/Cas9-mediated knockout of the *Chrm1* gene encoding M1 mAChR reduces REM sleep, which is a known AD feature (Liguori et al., 2016). Peng et al. (2019) designed sgRNA sequences for the production of loss-of-function alleles in mouse nAChR genes. They targeted candidate nAChR genes *in vivo* by creating herpes simplex virus (HSV) vectors delivering sgRNAs and Cas9 expression to mouse brain. This approach allowed to study the contribution of specific receptor subunits in cholinergic transmission.

## Development of New *in vitro* and *in vivo* AD Models Using Genome Editing Tools

Methods of genome editing have been applied to studies on the role of specific AD associated proteins, mutations in which definitely lead to the development of the disease. The *PSEN1* gene is located in chromosome 14 and encodes the PSEN1 protein, which is a part of a γ-secretase complex in the cellular membrane. This complex cleaves other membrane proteins of type I into peptides, and APP is the most well-known substrate of the enzyme. Presently, 185 mutations are known in this gene, most of them leading to a common molecular phenotype such as an increased Aβ42:Aβ40 ratio (Tanzi, 2012). Woodruff et al. (2013) used TALEN to introduce and study the role of mutations in the *PSEN1* gene in isogenic euploid induced pluripotent human stem cells (iPSCs). Data on the expression of some *PSEN1* alleles in differentiated cells allowed the authors to reveal that, even at a normal expression level, mutation in the gene impaired γ-secretase activity of PSEN1 in the respective type of cells but did not affect the other protein functions unrelated to this activity.

The CRISPR/Cas9 system is a suitable tool for generation of isogenic human iPSCs lines. This approach allows to simultaneous study of the effect of the presence of specific mutations introduced using gene editing tools in “diseased” and “healthy” cells with the same set of genes (Pires et al., 2016; Poon et al., 2016). The CRISPR/Cas9-based genome-editing framework was used for transduction human iPSCs with heterozygous and homozygous dominant early onset AD-causing mutations in APP [APP (Swe)] and PSEN1 [PSEN1 (M146V)]. After the induction of neuronal differentiation in those iPSCs, the derived cortical neurons displayed genotype-dependent disease-associated phenotypes reflected in altered Aβ metabolism. Thus, the presence of the APP (Swe) mutation resulted in elevated Aβ production in neural precursors and neurons whereas the PSEN1 (M146V) mutation shifted the Aβ42:Aβ40 ratio to the prevalence of Aβ42 in iPSCs already and in the next stages of neuronal differentiation (Paquet et al., 2016). Furthermore, CRISPR/Cas9-mediated deletion of *APP* in cortical neurons derived from iPSCs of patients with monogenic PSEN1 mutation supported a key role for proteolysis of the C-terminal of APP by γ-secretase in neuronal dysfunction in AD (Hung and Livesey, 2018).

Sigma-1 receptor (S1R) is an endoplasmic reticulum resident transmembrane protein, which is important for stability of mushroom spines in hippocampal neurons. The agonists of this receptor exhibit neuroprotective effects in cellular and animal models of AD (Maurice et al., 2019). Using CRISPR/Cas9, Ryskamp et al. (2019) have studied the involvement of S1R in the maintenance of mushroom spines stability in hippocampal neurons from wild-type or PSEN1-knock-in mice. They deleted endogenous S1R and substituted it with human S1R or its mutant forms. They found that the agonist of S1R pridopidine did not rescue mushroom spines in PSEN1-mutant cultures if mutant human S1R variants were expressed.

Sasaguri et al. (2018) applied CRISPR-Cas9-based base-editing technology, in which catalytically deactivated Cas9 (dCas9) or Cas9 nickase (nCas9) was fused with cytidine deaminases to convert C:G base pairs to T:A base pairs at target sites with a reduced rate of indel formation in the presence of sgRNAs, for production of multiple animal models with a number of distinct disease-related and disease-unrelated point mutations in the *PSEN1* and *APP* genes. For this purpose the authors injected RNA solutions containing several types of base editors and sgRNAs into the cytoplasm of C57BL/6J zygotes and then embryos at the 2-cell-stage were transferred to host ICR female mice. After that they studied the functional consequences of these mutations *in vivo*. Though the phenotypic features of these mutant mice did not significantly differ from those in wild type animals, they exhibited higher levels of Aβ42. In addition to *PSEN1*-P436S and *PSEN1*-P117L this approach allowed the authors to identify, a potential novel pathogenic mutation *PSEN1*-P436L that had not been previously reported.

Sortilin-related receptor L (SORL1) is a neuronal APOE receptor, the gene of which is located in chromosome 11 and predominantly expressed in the CNS. Lack of the APOE receptor SORL1 expression has been found in brain tissue of AD patients (Scherzer et al., 2004). Knockout of the gene encoding SORL1 using CRISPR/Cas9 was followed by the development of an AD-like phenotype in mice (Lin et al., 2018). Thus, SORL1^−/−^ mice had behavioral abnormalities and APP and Aβ expression in the brain, which were similar to those found in APP/PS1 mice used as a positive control. The authors suggested considering this strain of mice as a model of sporadic AD. Thus, the use of a genome editing approach gives us an opportunity not only to create new cellular or animal models of AD with face or construct validity allowing us to study some aspects of the pathogenesis, but also have predictive validity.

## Experimental Treatment of AD Using Genome Editing Tools

**Figure 1:**
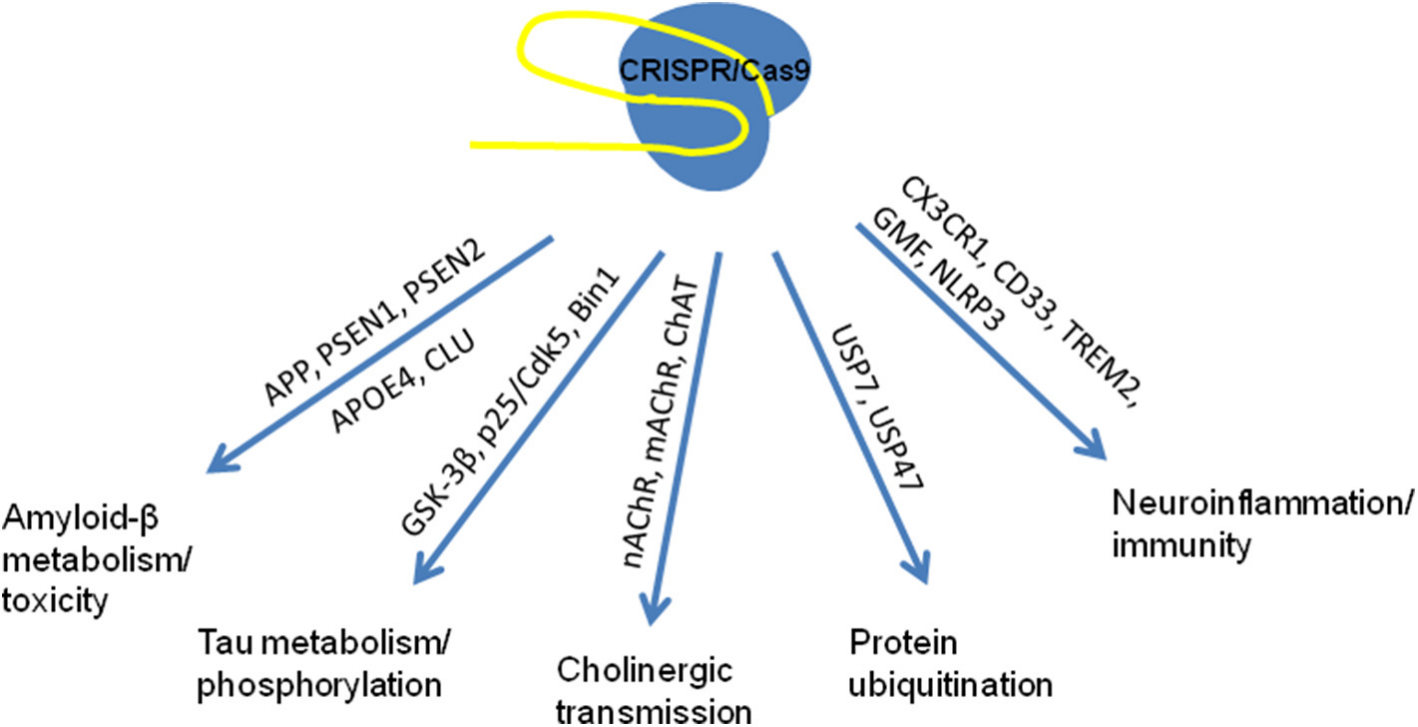
Known pathways associated with mutations in the genes involved in multifactor AD pathogenesis used as targets for genome editing in studies *in vitro* and *in vivo* (based on Robinson et al., 2017; Raikwar et al., 2019; Sims et al., 2020).

Application of pathway analysis methods to GWAS data from AD studies allowed to identify disease-relevant processes and to provide the convincing genetic evidence on pathways involved in etiology of AD (Sims et al., 2020). Some of these pathways and possible targets for therapeutic application of genome editing for correction of the functional consequences of well-known mutations are presented in Figure 1.

Presently, there are only a few studies have been performed to examine the therapeutic potential of genome editing technology in AD models. The main idea was to correct gene mutations associated with familial AD. Specifically, György et al. (2016) used CRISPR/Cas9 to edit the mutant APP(SWE) gene in fibroblasts from AD patients. Cell transfection with CRISPR plasmids and sgRNAs designed against the mutated or non-mutated sites resulted in the destruction of mutant and normal alleles of the gene, respectively, with high specificity. Deep sequencing did not reveal any random mutations on the normal allele with the gRNA targeting the mutated site or vice versa. Most modifications were deletions, though some insertions were also observed. These alterations decreased the production of Aβ40 and Aβ42. This group of authors also studied the possibility of editing the mutant APP(SWE) allele in primary neuronal culture from Tg2576 mice (György et al., 2018). For this purpose, neurons were transduced with two rAAV vectors carrying sgRNA and Cas9. A significant decrease in Aβ production was observed in the treated cell culture. These vectors were also injected into the hippocampus of adult APP(SWE) mutant mice, and targeted disruption of the mutant APP allele due to indel formations followed by frame shifts of the DNA sequence was observed.

As mentioned above, BACE1 which is also required for the production of Aβ peptides is another promising therapeutic target for AD treatment. Park et al. (2019) have studied whether CRISPR/Cas9-loaded nanocomplexes can efficiently target *Bace1* in the post-mitotic neurons of the adult 5XFAD mouse brain and APP knock-in AD mouse models. For this purpose, Cas9 and sgRNAs were loaded into an amphiphilic nanocomplex which allowed for efficient gene targeting in post-mitotic neurons *in vivo*. The authors reported that Cas9 nanocomplex-mediated targeting of BACE1 ameliorated Aβ-associated pathologies and cognitive deficits in both 5XFAD and *APP* knock-in mouse models of AD. A CRISPR/Cas9-based strategy was applied in cell and animal models to edit the *APP* gene close to the extreme C-terminus encoding the cleavage site for BACE-1 and reciprocally manipulate the amyloid pathway, attenuating APP-β-cleavage and Aβ production, while up-regulating neuroprotective APP-α-cleavage (Sun et al., 2019). APP N-terminus and compensatory APP-homologs remained intact, with no apparent effects on neurophysiology *in vitro*. Robust APP-editing was seen in human iPSC-derived neurons and mouse brains with no detectable off-target effects. This strategy limits APP and BACE-1 approximation probably due to the abrogation APP/BACE-1 convergence under this condition.

It is possible to decrease the production of toxic Aβ40/42 peptides via modification of γ-secretase activity, primarily mutant PSEN1 or its homolog PSEN2. It has been shown that it is possible to correct familial mutation in *PSEN2*-N141I in cholinergic neurons derived from iPSCs of control subjects or AD patients using the CRISPR/Cas9 system. CRISPR/Cas9 correction of the *PSEN2* point mutation not only normalized the Aβ42:Aβ40 ratio, but also abolished the electrophysiological deficit, restoring both the maximal number of spikes and spike height to the levels recorded in controls (Ortiz-Virumbrales et al., 2017). These data indicate the possible use of CRISPR/Cas9 as a potential therapeutic tool for the treatment of familial AD-associated mutations. On the other hand, iPSCs are considered a tool for modeling of AD pathology in a dish (Amin et al., 2019; Penney et al., 2020). Basal forebrain cholinergic neurons could be produced via directed differentiation of iPSCs derived from autologic fibroblasts of AD patients with known familial mutations (Ortiz-Virumbrales et al., 2017; Moreno et al., 2018). However, their further use for substitution of degenerated neurons in the brain remains questionable. Despite this, the recent successful application of autologous iPSCs generated from skin with corrected gene mutation for the treatment of sickle cell anemia in mice (Hanna et al., 2007) gives us a hope.

All studies mentioned above were directed to correction of known mutations, which are responsible for early-onset AD. The main factor, which is responsible for genetic predisposition to the development of late-onset AD, is *APOE4* genotype. A principal capability to convert the *APOE4* gene variant into *APOE3* in immortalized rat astrocytes, containing the mutant allele, has been demonstrated (Komor et al., 2016). The authors used a base editor (BE) for C->T exchange at codon 158. After nucleofection of these astrocytes with DNA encoding BE3 and appropriate sgRNA placing the target C at position 5 relative to a downstream PAM followed two-day incubation, the conversion of Arg158 to Cys158 in 58–75% of total DNA sequencing reads was observed. In the other study, iPSCs derived neurons from two different patients with APOE4/E3 genotypes were used (Wadhwani et al., 2019). APOE4/E3 neurons in culture exhibited increased APP processing, τ phosphorylation, and vulnerability to calcium deregulation compared to neurons derived from control patient's iPSCs. After CRISPR/Cas9-mediated editing, isogenic neuronal cells with APOE3/E3 were produced. Isogenic E4/E3 and E3/E3 neurons had comparable differentiation and survival rate, similar soma density, average neurite length per cell whereas E3/E3 neurons exhibited an increased number of neurite branch points per cell. E3/E3 neurons were more resistant to cytotoxins and reduced τ phosphorylation level but not amyloid processing. Taken together these data show not only the importance of APOE ε4 allele for the development of AD but also a possible pathway to protect neurons at least *in vitro*.

The presence of neurofibrillary tangles consisting of hyperphosphorylated τ-protein in the brain is a hallmark of AD; however, there are no mutations in the *MAPT* gene encoding this protein, which could be associated with familial AD forms. Most of well-known mutations in the *MAPT* gene were revealed in patients with frontotemporal dementia (FTD). Data from several studies performed in human iPSCs from patients with FTD associated with mutations in the *MAPT* gene were recently published. Aberrant Cdk5 activity results in activation of several pathological mechanisms of AD, including τ-protein hyperphosphorylation, due to accumulation of p25, an activator protein formed after proteolysis of p35 protein. To validate the role of p25/Cdk5 signaling pathway in the development of tauopathy, FTD-patient-derived iPSCs carrying the τ P301L mutation were used to generate P301L:Δp35KI isogenic iPSC lines, in which wild p35 was replaced with non-cleavable mutant Δp35 using CRISPR/Cas9 (Seo et al., 2017). These cell lines were used to create cerebral organoids with the reduced levels of phosphorylated τ due to the blockade of p25 production. Fong et al. (2013) used ZFN to correct the rare A152T mutation in the *MAPT* gene associated with increased risk of FTD and AD. After differentiation of human iPSCs to neurons, the cells had short, malformed processes, in which the point distribution of τ-protein and strong immunoreactivity of phosphorylated τ were observed. After correction via genome editing, these phenotypical signs disappeared. In the other study by Hallmann et al. (2017) neural precursors derived from human iPSCs with the N279K mutation in *MAPT* were used. The cells were transfected with a plasmid vector carrying sgRNA and Cas9 and single-stranded oligodeoxynucleotide, which was used as a template, to correct the mutation in exon 10. After genome sequencing in the prepared cell clones, the authors did not reveal any significant differences in the transcriptomes of the initial cells or the cells subjected to genome editing with CRISPR/Cas9 followed by HDR. The results of another study were more hopeful, because, using CRISPR/Cas9, the authors corrected the R406W mutation in *MAPT* in the cell line derived from human iPSCs of a patient with FTD with parkinsonism (Nimsanor et al., 2016). The sequencing revealed reparation of the mutant triplet without additional indel mutations in the site of the break made by CRISPR/Cas9. In addition, the cells maintained their pluripotency and normal karyotype.

## What is at the End of the AD Tunnel?

**Figure 2:**
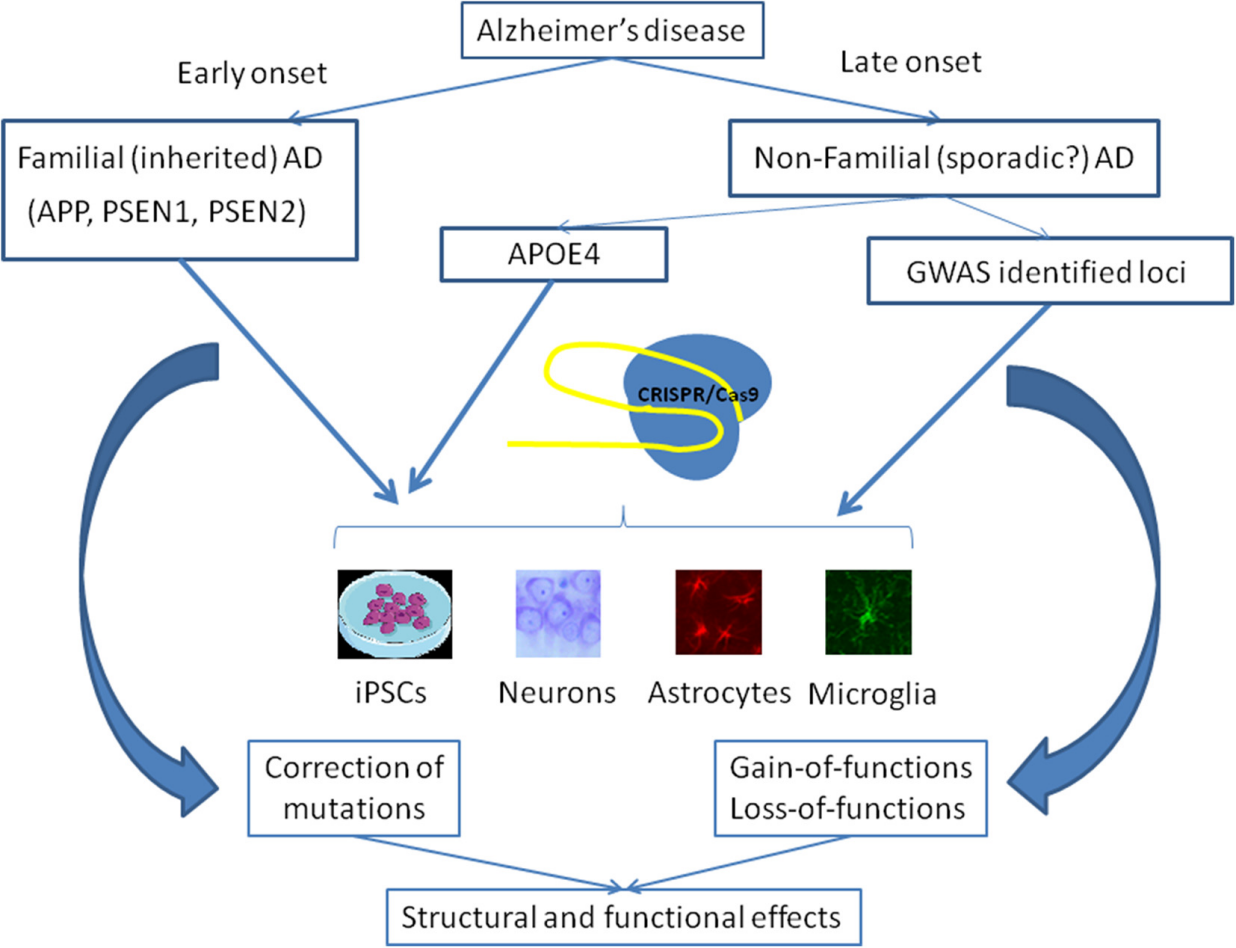
Main strategies of the use of CRISPR/Cas9 gene editing in the studies on Alzheimer's disease. Early-onset AD is associated with some known mutations in the *APP, PSEN1*, and *PSEN2* genes. CRISPR/Cas9 may be applied to correct these mutations in cells lines, including isogenic iPSCs, or different types of cells in the brain in order to examine structural and functional consequences of this therapy. APOE4 genotype predisposes to the development of late-onset AD and the correction of this genotype may be performed using CRISPR/Cas9. Moreover, several dozens of SNPs revealed in GWAS may be studied for their importance to the pathogenesis of late-onset AD as well as to normal functioning of the brain.

AD is a multifactorial disease, the main feature of which is dementia. AD is not a monogenic disease and the same clinical symptoms may be observed as a result of the presence of mutations in many genes. The association of these mutations with the progression of the disease is not fully understood; however, the involvement of many genes suggests convergence of their effects in functions. The way from GWAS to the development of new therapies represents a significant challenge for modern medicine. Methods of genome editing, specifically CRISPR/Cas9, may be used for simultaneous correction of multiple gene mutations, although the possibility of the application of this approach to the central nervous system remains uncertain. Figure 2 shows general purposes of application of the CRISPR system for investigation of AD. Firstly, genome editing is used for the correction of well-known mutations, which are responsible for the early-onset familial AD forms, and studies of the consequences of this treatment, as described above. Secondly, CRISPR/Cas9 is a suitable tool to study the consequences of the mutations in genes, which are not causative in order to reveal their role in brain physiology and pathophysiology of the late-onset AD forms. Thus, genome editing can be used to study whether the newly identified mutations can cause or act in combination with others to induce the development of a disease phenotype.

AD is a presenile or senile form of dementia, which is manifested when degeneration of brain structures becomes extensive. High probability of AD development is a specific feature of known familial forms of the disease. Thus, the question is what a period of ontogeny is suitable to correct the mutations? Although successful editing of a human embryo was reported three years ago (Ma et al., 2017) many ethical problems of this approach remain unresolved. These problems were raised in a recent discussion of *Nature* with Dr. D. Rebrikov, a Russian scientist who announced his experiments on gene editing in human eggs with the goal of altering genes that cause deafness, but who does not plan to implant gene-edited embryos until he gets regulatory approval (Cyranoski, 2019). The recent example of He Jiankui, a Chinese scientist who edited babies' genes and was jailed for three years for illegal practice, also demonstrates the acuity of the problem for society.

In addition to these ethical aspects of genome editing applications, it is important to answer many specific questions that stand in the way of practical use of CRISPR/Cas9 in medicine generally and for the treatment of AD in particular. One of the most important is the question of non-specific effects caused by the small size of the sgRNA binding site and the characteristics of Cas9 activity. For example, Sasaguri et al. (2018) in their study with a base editor for *PSEN1* also observed side effects in the *PSEN2* gene. Genetically determined forms of AD are dominant-negative, so editing should only affect the mutant allele, without affecting the healthy gene. The solution to this problem is quite difficult, since it requires either the identification of single-nucleotide polymorphisms in the mutant allele, or the artificial introduction of such polymorphisms, which could increase the specificity of sgRNA targeting.

The other major problem is the delivery of CRISPR/Cas components to cells. The large size of the CRISPR/Cas9 plasmid significantly limits the delivery of the structure to the cell both *in vitro* and *in vivo*. Delivery to the neurons of patient's brain after the onset of the disease with severe degenerative changes is apparently impractical, although as we mentioned above, Tuszynski et al. (2015) have used rLV-based vectors for direct delivery of the *NGF* gene into the brain of AD patients. Development of new technological solutions for CRISPR/Cas is very rapid. One recent design is that of Nanoblades, a protein-delivery vector-based on friend murine leukemia virus (MLV) that allows the transfer of Cas9-sgRNA ribonucleoproteins (RNPs) (Mangeot et al., 2019). Nanoblades deliver the RNPs cargo in a transient and rapid manner without delivering a transgene and can mediate knock-in in cell lines and primary cells including human iPSCs, human hematopoietic stem cells, and mouse bone-marrow cells when complexed with a repair template.

A possible alternative is an approach in which autologous fibroblasts or iPSCs can be modified *in vitro*, and then cells with successfully performed genome correction can be used to repopulate specific parts of the brain. Application of this approach has several advantages such as the absence of ethical questions and complete histocompatibility but also some disadvantages, including complex operation, low reprogramming efficiency, mutation predisposition, and tumorigenicity, also exist (Zhang et al., 2020). Furthermore, AD, particularly in advanced stages, is a multifocal disease with pathological alterations in cortical and subcortical structures and even more expressed decrease in structural connectivity revealed by the MRI methods (Palesi et al., 2016). Despite many unsolved problems and challenges related to the use of reprogrammed autologous cells, stem cells, or iPSCs, the data indicate that this therapy is still a prospective method for AD treatment.

Presently increasing attention is paid to innovative technologies for the treatment of neurodegenerative diseases, particularly of AD, because of the prevalence of these disorders in the elderly population. Genome editing is a rapidly developing field of research with significant progress made in the last decade. However, genome editing technology seems unlikely to come out of laboratories in the near future. The methods of application of genome editing in the brain become easier but the pathogenesis of AD remains poorly understood even for familial cases. Probably, we shall need to divide gene-mutation-associated AD and its sporadic forms in order to understand the specific role of genetic and epigenetic factors and create a new concept of this mysterious disease. In any case the development of new methods for investigation and treatment will include technologies based on genome editing.

## Author Contributions

MS collected, analyzed, and interpreted literature data, wrote the draft, and final version of the manuscript.

## Conflict of Interest

The author declares that the research was conducted in the absence of any commercial or financial relationships that could be construed as a potential conflict of interest.
